# Protective role of endothelial calpain knockout in lipopolysaccharide-induced acute kidney injury via attenuation of the p38-iNOS pathway and NO/ROS production

**DOI:** 10.1038/s12276-020-0426-9

**Published:** 2020-04-28

**Authors:** Zhifeng Liu, Jingjing Ji, Dong Zheng, Lei Su, Tianqing Peng, Jing Tang

**Affiliations:** 1 Department of Critical Care Medicine, General Hospital of Southern Theatre Command of PLA, 510010 Guangzhou, China; 2 Critical Illness Research Center, Lawson Health Research Institute, Departments of Medicine and Pathology, Western University, London, ON N6A 4G5 Canada; 3 Guangdong Provincial Key Laboratory of Proteomics; School of Basic Medical Sciences, Southern Medical University, 510515 Guangzhou, China; 4 Key Laboratory of Hot Zone Trauma Care and Tissue Repair of PLA, General Hospital of Guangzhou Military Command, 510010 Guangzhou, China; 5 Department of Anesthesia, Affiliated Hospital of Guangdong Medical University, Zhanjiang, Guangdong China; 6 Department of Anesthesiology, Nanfang Hospital, Southern Medical University, 510515 Guangzhou, Guangdong China

**Keywords:** Bacterial infection, Acute kidney injury

## Abstract

To explore the role of calpain and its signaling pathway in lipopolysaccharide (LPS)-induced acute kidney injury (AKI), animal models of endotoxemia were established by administration of LPS to mice with endothelial-specific Capn4 knockout (TEK/Capn4^−/−^), mice with calpastatin (an endogenous calpain inhibitor) overexpression (Tg-CAST) and mice with myeloid-specific Capn4 knockout (LYZ/Capn4^−/−^). Mouse pulmonary microvascular endothelial cells (PMECs) were used as a model of the microvascular endothelium and were stimulated with LPS. Renal function, renal inducible nitric oxide synthase (iNOS) and endothelial NOS (eNOS) expression, cellular apoptosis, plasma and renal levels of NO and reactive oxygen species (ROS), and phosphorylation of mitogen-activated protein kinase (MAPK) family members (p38, ERK1/2, and JNK1/2) were examined. Moreover, a calpain inhibitor, calpastatin overexpression adenoviruses and MAPK inhibitors were used. Significant renal dysfunction was induced by LPS stimulation, and recovery was observed in TEK/Capn4^−/−^ and Tg-CAST mice but not in LYZ/Capn4^−/−^ mice. Endothelial Capn4 knockout also abrogated the LPS-induced increases in renal iNOS expression, caspase-3 activity and apoptosis and plasma and renal NO and ROS levels but did not obviously affect renal eNOS expression. Moreover, LPS increased both calpain and caspase-3 activity, and only the expression of iNOS in PMECs was accompanied by increased phosphorylation of p38 and JNK. Inhibiting calpain activity or p38 phosphorylation alleviated the increased iNOS expression, NO/ROS production, and cellular apoptosis induced by LPS. These results suggest that endothelial calpain plays a protective role in LPS-induced AKI by inhibiting p38 phosphorylation, thus attenuating iNOS expression and further decreasing NO and ROS overproduction-induced endothelial apoptosis.

## Acute kidney injury: enzyme block could combat failure

Therapies that inhibit the enzyme calpain could alleviate the effects of acute kidney injury according to researchers in China and Canada. Acute kidney injury is induced by endotoxemia, in which changes in the permeability of the intestine allow lipopolysaccharides (LPS) to pass from gut bacteria into the bloodstream. Calpain is known to be active during this process. Zhifeng Liu at the General Hospital of Guangzhou Military Command and co-workers induced endotoxemia in various mouse models by injecting them with LPS. The LPS induced significant kidney dysfunction and cell death, but these were alleviated in mice that were genetically modified to block calpain activity in the blood vessel lining, and in mice that overexpressed calpastatin, a calpain inhibitor. Blocking calpain reduces the expression of nitric oxide synthases that damage endothelial cells.

## Introduction

Acute kidney injury (AKI) is one of the major components of multiorgan dysfunction syndrome (MODS). Sepsis is a life-threatening condition of organ dysfunction caused by a dysregulated host response to infection^1^ and the leading cause of AKI, accounting for nearly half of all AKI events^2^. Septic AKI is associated with poor outcomes, including a prolonged duration of ICU stay and increased mortality^3^. Multiple factors in sepsis, including hemodynamic instability, drug toxicity, microcirculation alterations, endothelial dysfunction, and others, can lead to AKI. Under normal conditions, the kidney receives 20% of cardiac output, and an abundance of capillary networks are responsible for glomerular filtration. Vascular endothelial cells play a crucial role in maintaining the homeostasis and integrity of the endothelial barrier in the kidney. During sepsis, endothelial cells are exposed to various stimuli, such as endotoxins and inflammatory cytokines. The endothelium undergoes structural changes, including endothelial cell death and loss of cell-cell contact, resulting in increased endothelial permeability. Disruption of the endothelial barrier leads to the leakage of albumin and large endogenous molecules into the urine. Moreover, endothelial dysfunction enhances leukocyte extravasation, which leads to leukocyte transmigration to the renal interstitium^4^. Subsequently, leukocytes exit the capillaries and directly induce tubular cell injury via inflammatory and oxygen stress responses^5^. Although endothelial dysfunction has been found to play a central role in septic AKI, targeted preventive and therapeutic measures are still lacking due to the unclear pathological mechanisms underlying this condition.

Calpains are calcium-dependent cysteine proteases that are ubiquitously expressed in mammals. Calpain-1 and calpain-2 are the most highly expressed members in tissues and are involved in many physiological and pathological processes. Structurally, these molecules exist as heterodimers composed of large catalytic subunit of 80 kD and a small regulatory subunit of 30 kD^6^. The small regulatory subunit is encoded by the *Capn4* gene^7^. Calpain is involved in many physiological and pathological processes because of its proteolytic activity^8^. The substrates of calpain vary and include signal transduction proteins and transcription factors^9^, indicating that calpains are important in a wide range of calcium-regulated cellular functions. Calpastatin is the predominant endogenous inhibitor of calpain and limits the proteolysis of calpain substrates. Calpain activation has been shown to induce hepatic inducible nitric oxide synthase (iNOS) during lipopolysaccharide (LPS) stimulation^10^. Our previous research found that calpain activity was correlated with an increase in reactive oxygen species (ROS) production and peroxynitrite formation^11,12^. Endothelial cells are sensitive to these vasoactive substances, thereby regulating microcirculation and glomerular filtration^13^. These results prompted us to determine whether calpain activity is associated with septic AKI and whether endothelial calpain-targeted treatment could be a therapeutic strategy.

In this study, to investigate the role and potential mechanism of endothelial cell calpain in LPS-induced renal dysfunction, we established animal models of endotoxemia in mice with endothelial-specific Capn4 knockout (KO) (TEK/Capn4^−/−^), mice with calpastatin overexpression (Tg-CAST) and mice with myeloid-specific Capn4 knockout (LYZ/Capn4^−/−^), and we established an in vitro model using pulmonary microvascular endothelial cells (PMECs). We demonstrated that endothelial calpain knockout plays a protective role in LPS-induced AKI by inhibiting p38 phosphorylation and attenuating endothelial injury induced by iNOS expression and NO/ROS production.

## Materials and methods

### Animals

Breeding pairs of C57BL/6 mice were purchased from The Jackson Laboratory (Sacramento, CA USA), and transgenic Tg-CAST mice were kindly provided by Dr. Laurent Baud (the Institut National de la Santé et de la Recherche Médicale, Paris, France)^14^. Transgenic mice with endothelial-specific Capn4 knockout (TEK/Capn4^−/−^) and myeloid-specific Capn4 knockout (LYZ/Capn4^−/−^) were purchased from The Jackson Laboratory, and a breeding program was implemented at our animal care facilities^12^.

All animals were provided food and water ad libitum and were housed in a temperature-controlled and humidity-controlled facility with 12-hour (h) light and dark cycles. All animals were used in accordance with the Canadian Council on Animal Care guidelines, and all experimental protocols were approved by the Animal Use Subcommittee at the University of Western Ontario.

### Establishment of endotoxemia animal models

Animal models of endotoxemia were established by administration of LPS (4 mg/kg intraperitoneally (i.p.), Sigma) or saline as the control, according to our previous study^15^. After 18 h, the animals were euthanized and exsanguinated by cardiac puncture. Blood was processed to obtain plasma according to the method published by Madorin et al.^16^. In addition, urine and kidney tissues were collected for further examination.

### PMEC culture and treatments

PMECs were isolated from adult C57BL/6 mice and cultured as previously described^17^. All PMECs were used for this study within 5 passages. Calpain inhibitor III, SB203580, PD98059, and SP600125 were purchased from Sigma, Calbiochem or Life Technologies. All inhibitors were administered 1 h before other treatments. Cells were treated with the indicated concentration of LPS for 18 h or pretreated with the above inhibitors for 1 h followed by stimulation with LPS (1 µg/ml) for an additional 18 h.

### Measurement of renal function

Blood urea nitrogen (BUN) was assayed in accordance with the kit manufacturer’s instructions (BioAssay Systems, Hayward, CA). Mouse urine was collected before the animals were euthanized. Urinary albumin was measured in accordance with the manufacturer’s instructions (Bethyl Laboratories, Montgomery, TX).

### Caspase-3 activity

Tissue and cellular caspase-3 activity was measured using a caspase-3 fluorescent assay kit according to the manufacturer’s protocol (BIOMOL Research Laboratories), as previously described^18^.

### DNA fragmentation

Cells were prelabeled with BrdU for 24 h before other treatments. DNA fragmentation was measured using a cellular DNA fragmentation ELISA kit (Roche Applied Science) according to the manufacturer’s instructions^15^.

### In situ detection of apoptotic cells

A terminal deoxynucleotidyl transferase-mediated dUTP nick-end labeling (TUNEL) assay was used. Kidney tissue was fixed in 10% formalin and embedded in paraffin. Fixed kidney tissues were cut into 3-mm-thick blocks. The tissue blocks were embedded in paraffin and cut into 5-mm slices. After deparaffinization (using xylene and ethanol dilutions) and rehydration, the sections were subjected to TUNEL with an ApopTag Peroxidase In Situ Apoptosis Detection Kit (Chemicon, CA, USA), as described in previous studies^19^. Apoptotic cell death was quantitatively analyzed by counting the TUNEL-positive cells in 10 randomly selected fields at 200× magnification. The results are presented as the number of TUNEL-positive cells per 200× magnification field.

### Calpain activity

Calpain activity was assessed using the fluorescent substrate N-succinyl-LLVY-AMC (Cedarlane Laboratories, Burlington, Ontario, Canada), as described in our previous study^15^.

### Adenoviral infection of PMECs

Cultured PMECs were infected with adenoviral vectors containing the rat calpastatin gene (Ad-CAST, University of Buffalo, USA) or hemagglutinin (HA) (Ad-HA, Vector Biolabs) as the control at a multiplicity of infection of 10 plaque-forming units (PFU)/cell. Adenovirus-mediated gene transfer was performed as previously described^20^. All experiments were performed after 24 h of adenoviral infection.

### Western blot analysis

Protein samples were extracted from kidney tissue or cultured PMECs. Equal amounts of protein were subjected to SDS-PAGE. Binding of the primary antibody was then detected using peroxidase-conjugated secondary antibodies (goat anti-rabbit IgG-HRP, Bio-Rad Laboratories) and enhanced chemiluminescence (Amersham), and the band densities were quantified via densitometry. The antibody source and the dilution used were as follows: rabbit anti-calpain1 and 2, anti-phospho-p38, anti-phospho-JNK1/2, anti-phospho-ERK1/2, anti-total p38, anti-total JNK1/2, anti-total ERK1/2, anti-iNOS, and anti-eNOS antibodies (all at a 1:1000 dilution, Cell Signaling). Rabbit anti-GAPDH (1:1000 dilution, Santa Cruz) was used as the internal control.

### Measurement of NO and ROS production

NO production in plasma, tissue lysates and cell culture media was measured by using a commercial kit (Cayman Chemical Company) according to the manufacturer’s instructions. Briefly, 20 μl of each sample was incubated with a nitrate reductase mixture for 1 h at room temperature. The NO level was measured by detecting the fluorescent product 1(H)-naphthotriazole formed from the reaction between nitrite and 2,3-diaminonaphthalene.

The production of ROS in plasma and tissue lysates was measured using the ROS-sensitive dye 2′,7′-dichlorodihydrofluorescein diacetate (DCF-DA) as an indicator (Molecular Probes), as described in our previous study^21^.

### Real-time quantitative polymerase chain reaction (qPCR)

Analysis of iNOS and eNOS mRNA was conducted by real-time RT–PCR. Total RNA was extracted from kidney tissue using TRIzol reagent (Life Technologies Inc., Burlington, Ontario, Canada) according to the manufacturer’s instructions. Extracted RNA was reverse transcribed into cDNA by a reverse transcription kit (Toyobo CO., LTD, Osaka, Japan). Real-quantitative PCR was performed to analyze the mRNA expression of mouse iNOS, eNOS and GAPDH by SYBR Green I dye chemistry (Toyobo CO., LTD, Osaka, Japan). SYBR Green dye chemistry uses SYBR Green dye to detect PCR products by binding to double-stranded DNA formed during PCR. As the PCR progresses, more amplicons are created, with increasing fluorescence intensity that is proportional to the amount of PCR product produced. By measuring the threshold cycle (Ct), which is the intersection of an amplification curve and a threshold line, the relative mRNA expression of iNOS and eNOS was calculated by the 2^−ΔCt^ method. The following primers were used to amplify mouse iNOS, eNOS and GAPDH mRNA^22^: iNOS, 5’-ACA GGA GAA GGG GAC GAA CT-3’ (forward) and 5’-GGC TGG ACT TTT CAC TCT GC-3’ (reverse); eNOS, 5’-GAC CCT CAC CGC TAC AAC AT-3’ (forward) and 5’-CTG GCC TTC TGC TCA TTT TC-3’ (reverse); and GAPDH, 5’-AAA GGG CAT CCT GGG CTA CA-3’ (forward) and 5’-CAG TGT TGG GGG CTG AGT TG-3’ (reverse).

### Statistical analysis

All data are presented as the means ± SD. Differences between two groups were compared by an unpaired Student’s *t*-test. For multigroup comparisons, ANOVA followed by Student-Newman-Keuls test was performed. A value of *P* < 0.05 was considered statistically significant.

## Results

### LPS-induced renal injury was alleviated in mice with *Capn4* knockout

**Fig. 1:**
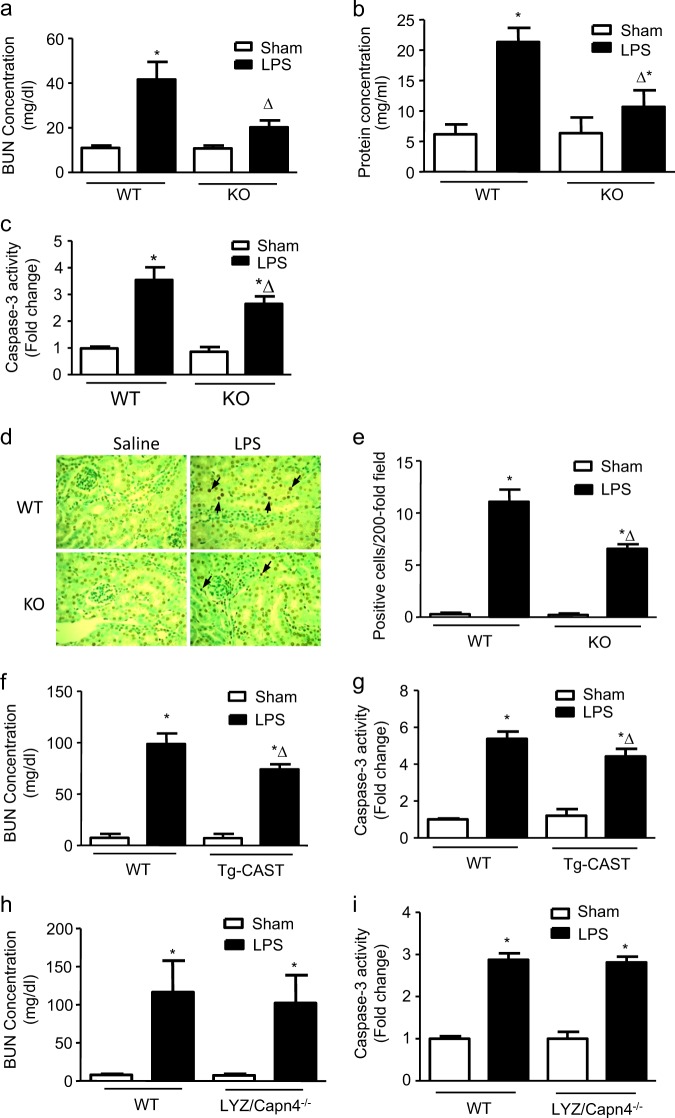
Role of endothelial-specific Capn4 knockout in LPS-induced kidney injury. Wild-type (WT) and endothelial-specific Capn4 knockout (KO) mice were stimulated with LPS (i.p., 4 mg/kg) for 18 h. The plasma BUN concentration (**a**), urinary protein levels (**b**), and renal caspase-3 activity (**c**), and TUNEL staining (**d**; the arrows show apoptotic cells) were assessed. Quantitative results of the number of TUNEL-positive cells per ×200 magnification field in kidney tissues are shown (**e**). In addition, mice with calpastatin overexpression (Tg-CAST) and myeloid-specific Capn4 knockout (LYZ/Capn4^−/−^) were also treated with LPS, and changes in kidney injury were assessed. BUN concentrations in plasma (**f** and **h**) and caspase-3 activity (**g** and **i**) in kidney tissue were detected. *P < 0.05 vs. the WT Sham group; ∆*P* < 0.05 vs. the WT LPS group, *n* = 6–8.

The endotoxemia models were established by intraperitoneal injection of LPS (4 mg/kg), and the plasma BUN concentration and urinary protein levels were measured to assess renal dysfunction. The plasma BUN concentration and urinary protein levels were significantly increased 18 h after LPS administration (Fig. 1a and b), indicating that LPS induced significant renal dysfunction. In addition, caspase-3 activity and the number of TUNEL-positive cells were significantly increased in renal tissues (Fig. 1c–e), suggesting that renal dysfunction developed beyond functional injury to organ damage.

Calpain has been found to be involved in the development of the inflammatory process^14^, and endothelial dysfunction plays a crucial role in sepsis-related organ dysfunction. To determine the effect of endothelial calpain on LPS-induced renal injury, we utilized mice with endothelial-specific knockout of Capn4, which encodes the small subunit of calpain-1 and calpain-2 (TEK/Capn4^−/−^). Compared with wild-type mice, TEK/Capn4^−/−^ mice exhibited significantly decreased plasma BUN concentrations and urinary protein levels 18 h after LPS injection (Fig. 1a and b). Renal cell apoptosis was also decreased in knockout mice, as evidenced by decreased caspase-3 activity and the proportion of TUNEL-positive cells (Fig. 1c–e).

Calpastatin is an endogenous inhibitor of calpain and globally inhibits calpain-1 and calpain-2 activity. To further confirm the effect of calpain on LPS-induced renal injury, mice with calpastatin overexpression (Tg-CAST) were treated with the same dose of LPS, and the plasma BUN concentration and caspase-3 activity were measured. The results were similar to those in TEK/Capn4^−/−^ mice; endotoxemic Tg-CAST mice exhibited reduced plasma BUN concentrations and decreased caspase-3 activity in renal tissue (Fig. 1f and g). However, this protective effect was not observed in mice with myeloid-specific Capn4 knockout (LYZ/Capn4^−/−)^ (Fig. 1h and i), indicating that endothelial calpain plays a dominant role in LPS-induced renal injury.

VIn conclusion, LPS injection induced significant renal dysfunction and cell apoptosis, which were alleviated when endothelial calpain activity was abolished.

### Calpain activation was involved in LPS-induced PMEC apoptosis

**Fig. 2:**
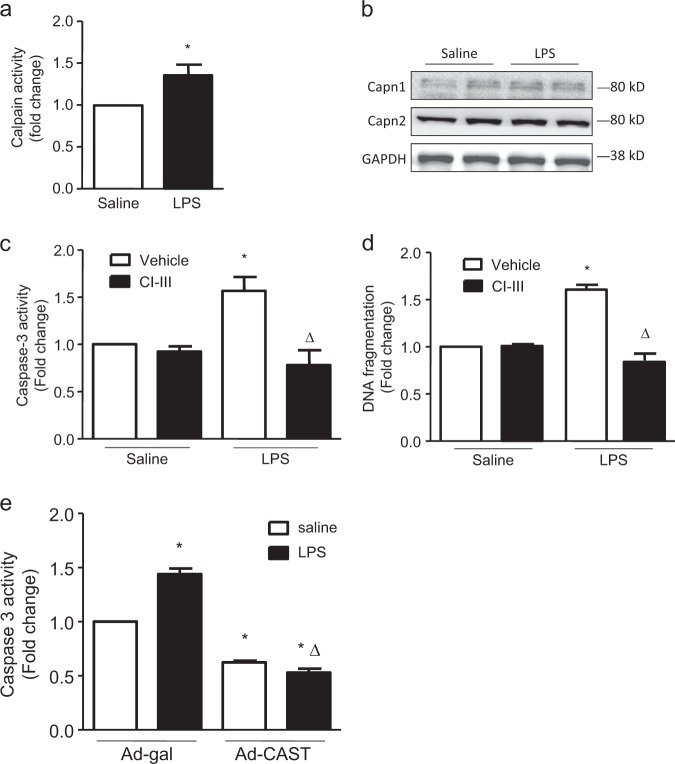
Role of calpain in LPS-induced PMEC apoptosis. PMECs were treated with calpain inhibitor III (CI-III, 5 µmol/l) for 1 h and then stimulated with LPS (1 µg/ml) for 18 h. Calpain activity (**a**) and the expression levels of calpain1 and calpain2 (**b**) were measured. Caspase-3 activity (**c**) and DNA fragmentation (**d**) were examined. Calpastatin overexpression adenoviruses (Ad-CAST) and Ad-gal were used as the control to infect PMECs before LPS stimulation, and caspase-3 activity (**e**) was examined. The data are presented as the means ± SD of 3 independent experiments. *P < 0.05 vs. the Sham vehicle/saline group. ∆*P* < 0.05 vs. the LPS vehicle/saline group.

To further clarify the role of calpain in endothelial cells, we utilized PMECs as an in vitro model. After LPS stimulation, calpain activity was increased significantly (Fig. 2a), but no changes were observed in the protein expression of either calpain-1 or calpain-2 (Fig. 2b). In addition, LPS treatment induced an increase in caspase-3 activity and DNA fragmentation, indicating cell apoptosis, and this effect was prevented by either administration of CI-III, an inhibitor of calpain activity (Fig. 2c and d), or overexpression of calpastatin, the endogenous inhibitor of calpain (Fig. 2e). These results suggest that calpain is involved in LPS-induced PMEC apoptosis.

### Capn4 knockout prevented LPS-induced apoptosis by inhibiting the phosphorylation of p38 mitogen-activated protein kinase (MAPK)

**Fig. 3:**
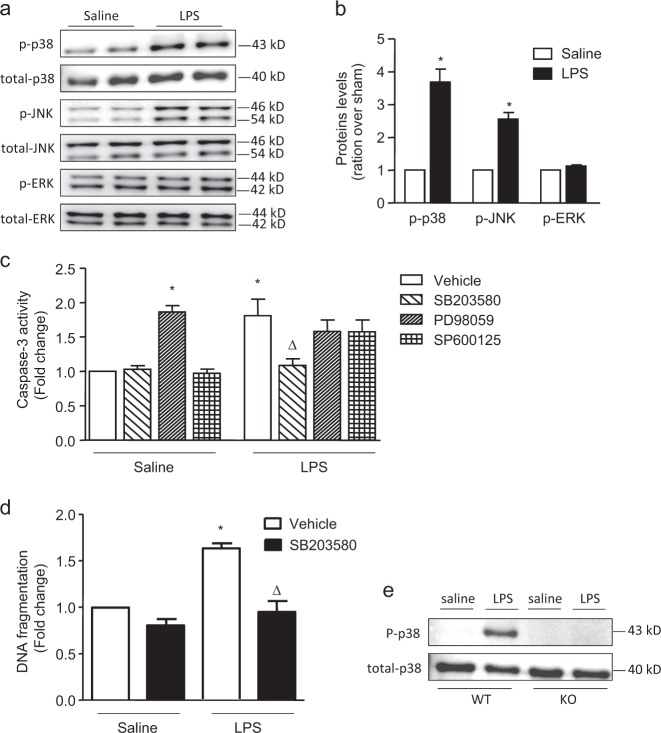
Role of MAPK family members in the apoptosis of LPS-stimulated PMECs. PMECs were stimulated with LPS (1 µg/ml) for 18 h. **a** Expression levels of total and phosphorylated p38, JNK (46 and 54 kDa, respectively) and ERK1/2 (42 and 44 kDa, respectively) were determined by western blot analysis. **b** The corresponding bands were quantified via densitometry (phospho/total protein, presented as fold changes with respect to Sham). In addition, PMECs were treated separately with MAPK inhibitors (10 µmol/l SB203580, 10 µmol/l PD98059 and 20 µmol/l SP600125) for 1 h and were then stimulated with LPS (1 µg/ml) for an additional 18 h. Caspase-3 activity (**c**) and DNA fragmentation (**d**) were assessed. **e** Changes in p38 phosphorylation in LPS-treated mice with endothelial-specific Capn4 knockout (KO) were determined by western blot analysis, and a representative image is shown. The data are presented as the means ± SD of 3 independent experiments. *P < 0.05 vs. the Sham vehicle group. ∆*P* < 0.05 vs. the LPS vehicle group.

Our previous study found that calpain is involved in cell apoptosis by regulating the phosphorylation of MAPKs^17^. To explore the mechanism of calpain in LPS-induced endothelial apoptosis, we measured the phosphorylation of MAPK family members, including p38, ERK and JNK, by western blotting. LPS treatment increased the phosphorylation of p38 and JNK but not ERK (Fig. 3a and b). We further explored the roles of MAPKs in LPS-induced PMEC apoptosis by treating cells separately with inhibitors of p38, JNK and ERK. Caspase-3 activity was decreased in the group that was treated with SB203580, an inhibitor of p38 phosphorylation (Fig. 3c). Similarly, SB203580 treatment decreased LPS-induced DNA fragmentation in PMECs (Fig. 3d). Notably, in TEK/Capn4^−/−^ mice, p38 phosphorylation was significantly suppressed (Fig. 3e). Collectively, these results suggest that the protective effect of Capn4 knockout is dependent on the decreased phosphorylation of p38 MAPK.

### Endothelial cell Capn4 deficiency reduced renal NO and ROS production in endotoxemic mice by inhibiting iNOS expression

**Fig. 4:**
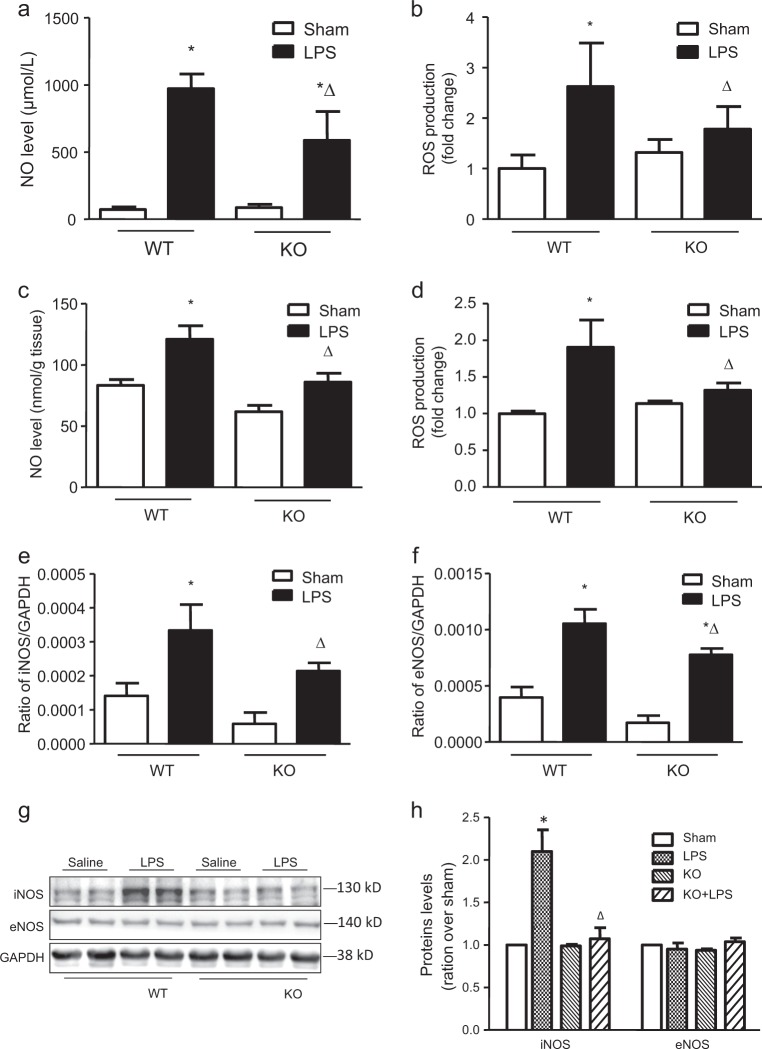
Changes in NO and ROS concentrations and the iNOS and eNOS mRNA and protein levels in the kidneys of mice with endothelial-specific Capn4 knockout (KO) that were treated with LPS (i.p., 4 mg/kg) for 18 h. NO and ROS concentrations in plasma (**a** and **b**) and kidney tissue (**c** and **d**) were measured. Real-time PCR was used to measure iNOS (**e**) and eNOS (**f**) mRNA levels, and western blotting was used to measure iNOS and eNOS protein levels (**g**). **h** The corresponding bands on the western blot were quantified via densitometry (protein/GAPDH, presented as fold changes with respect to Sham). *P < 0.05 vs. the WT Sham group; ∆*P* < 0.05 vs. the WT LPS group, *n* = 6–8.

NO and ROS are involved in the pathological process of renal injury^23^. Our previous research showed that inhibiting calpain by overexpressing calpastatin decreased NO production in a model of diabetes^11^. In this study, we further investigated the effect of calpain on NO levels and ROS production in a model of endotoxemia in TEK/Capn4^−/−^ mice. Compared with wild-type mice, TEK/Capn4^−/−^ mice exhibited decreased NO levels and ROS production both in plasma (Fig. 4a and b) and renal tissue (Fig. 4c and d). Since the production of NO and ROS is related to NOS, we measured the mRNA and protein levels of inducible NOS (iNOS) and endothelial NOS (eNOS) in kidney tissue (Figs. 4e and f). The mRNA levels of both iNOS and eNOS were increased in kidney tissue from endotoxemic mice, and endothelial Capn4 knockout suppressed this upregulation (Figs. 4e and f). However, the protein expression of only iNOS was significantly increased, and this upregulation was decreased in TEK/Capn4^−/−^ mice (Figs. 4g and h). These results indicate that Capn4 deficiency in endothelial cells reduces renal NO and ROS production in endotoxemic mice by inhibiting iNOS expression.

### Inhibition of calpain activity and p38 phosphorylation decreased iNOS expression and NO production in PMECs

**Fig. 5:**
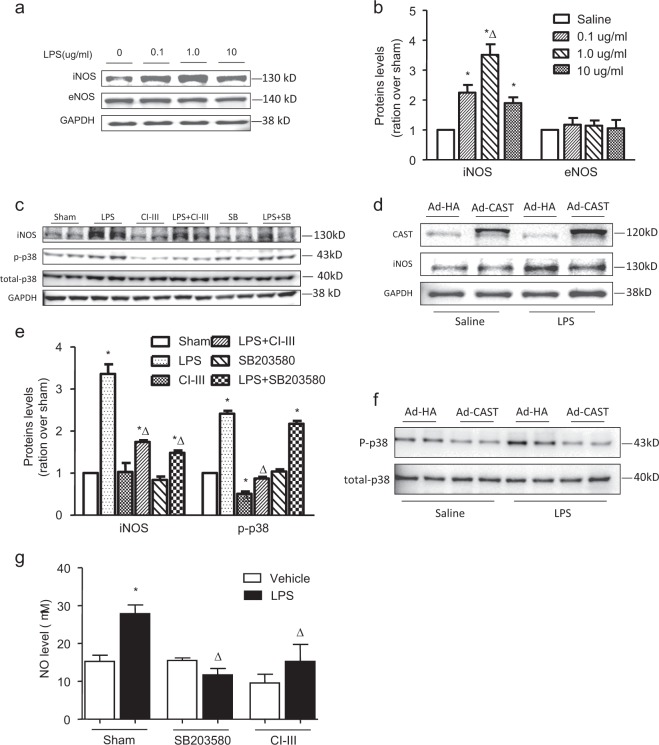
Changes in iNOS expression and the role of calpains in p38 activation, LPS-induced iNOS expression and NO production in LPS-stimulated PMECs. PMECs were stimulated with different doses of LPS (0.1–10 µg/ml) for 18 h, and iNOS expression (**a**) was measured. **b** The corresponding bands on the western blot were quantified via densitometry (protein/GAPDH, presented as fold changes with respect to Sham). Then, PMECs were treated with calpain inhibitor III (CI-III, 5 µmol/l) or SB203580 (10 µmol/l) for 1 h and were then stimulated with LPS (1 µg/ml) for 18 h. **c** The expression levels of iNOS and total and phosphorylated p38 were measured by western blot analysis. **d** The corresponding bands were quantified via densitometry (p-p38/total p38 or iNOS/GAPDH, presented as fold changes with respect to Sham). PMECs were infected with calpastatin overexpression adenovirus (Ad-CAST) or control adenovirus (Ad-HA) for 24 h and were then stimulated with LPS (1 µg/ml) for 18 h. **e** The expression levels of calpastatin, iNOS and GAPDH were measured by western blot analysis. **f** The level of phosphorylated p38 was determined by western blot analysis. **g** NO production in the supernatant was measured. The data are presented as the means ± SD of 3 independent experiments. *P < 0.05 vs. the Sham vehicle group. ∆*P* < 0.05 vs. the LPS vehicle group.

To clarify the relationship among calpain, p38 MAPK and iNOS, LPS-treated PMECs were utilized as an in vitro model. Western blot analysis showed that treatment with 1 μg/ml LPS induced significant upregulation of iNOS expression in PMECs, although no significant alteration in eNOS expression was observed (Figs. 5a and b). LPS-induced upregulation of iNOS was suppressed in cells that were pretreated with the calpain inhibitor or p38 phosphorylation inhibitor (Fig. 5c and d); moreover, NO levels were decreased (Fig. 5g). Similar results were found in the cells overexpressing calpastatin (Figs. 5e and f). Collectively, these results show that calpain activity and p38 phosphorylation are involved in LPS-induced production of NO by affecting the expression of iNOS.

## Discussion

**Fig. 6:**
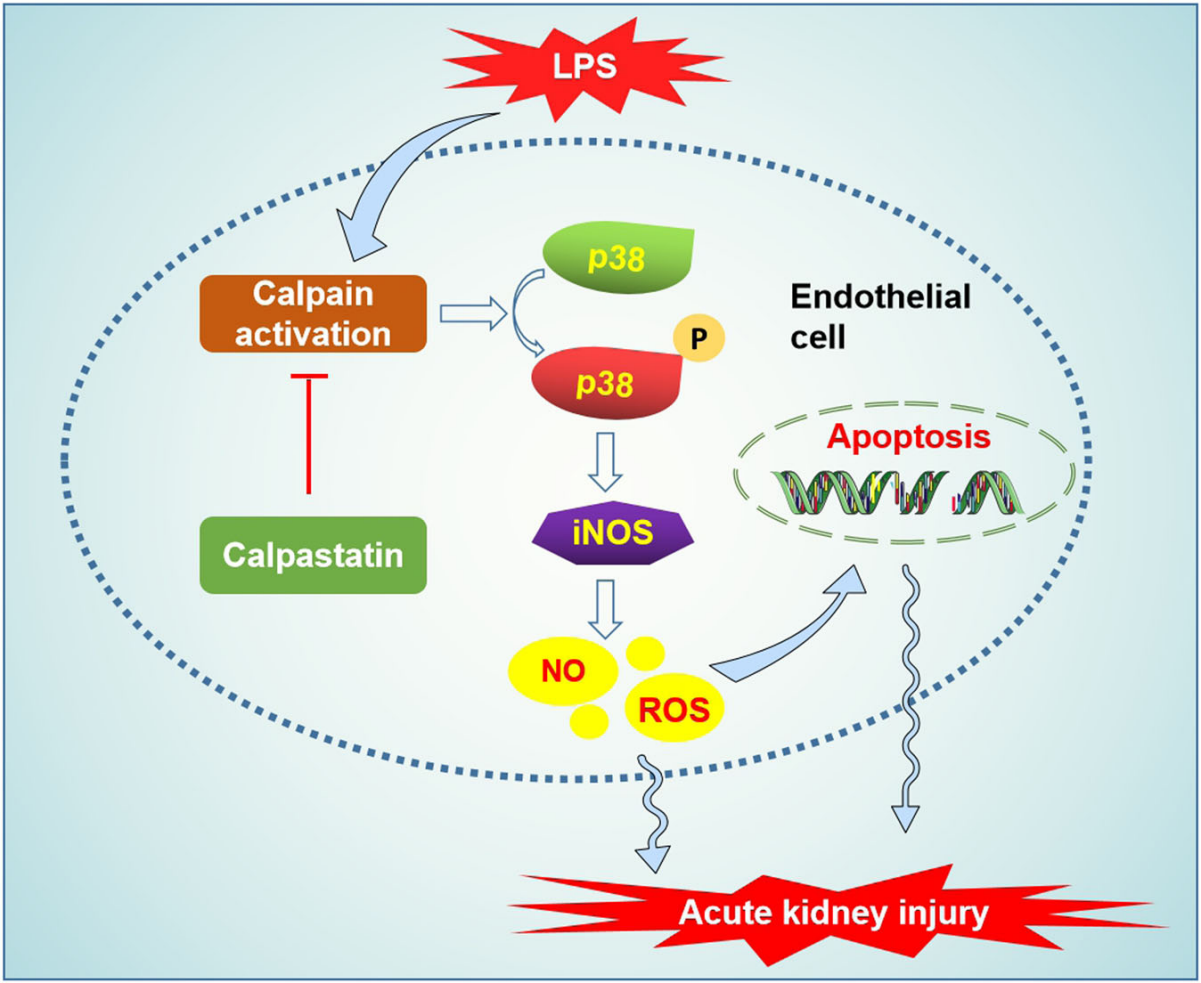
Schematic showing the signaling pathway involved in LPS-induced AKI in endotoxemic mice. LPS activates p38 MAPK via calpain activation, which increases endothelial iNOS expression and NO/ROS production, which in turn leads to endothelial apoptosis through the caspase 3 pathway, thus inducing AKI in endotoxemic mice.

This study is the first to report that endothelial cell-specific Capn4 knockout reduced LPS-induced renal dysfunction and that this protective effect may be attributed to attenuated apoptosis of endothelial cells via inhibition of p38 phosphorylation. Furthermore, inhibition of p38 phosphorylation reduced LPS-induced iNOS upregulation and further decreased the production of NO and ROS both in vivo and in vitro. These findings highlight a critical role of endothelial cell calpain in septic AKI (Fig. 6).

Calpain activation exerts a proapoptotic effect in mice with sepsis and in LPS-treated cultured cells, including cardiomyocytes and endothelial, diaphragm and skeletal muscle cells^16,17,24,25^. Since calpain is a protease, its proapoptotic effect is attributable mainly to its degradative properties. Calpain partially cleaves some apoptosis-related proteins, including caspase-3, caspase-9 and Bcl-2, which might activate or inactivate putative substrates^26,27^. The results of this study suggest that the proapoptotic role of calpain is related to caspase-3 activation, since Capn4 knockout abolished caspase-3 activation and DNA fragmentation in kidney tissue. In addition, we found that this proapoptotic effect was related to the phosphorylation of p38, similar to the findings in our previous study^17^. MAPKs are a family of serine/threonine kinases, and the ERK, JNK and p38 pathways are the three classical MAPK pathways in mammals and mediate various cellular processes, including cell proliferation, apoptosis, and stress responses^28^. Here, we found that phosphorylation of only p38 was involved in LPS-induced PMEC apoptosis. Although increased phosphorylation of JNK was observed in LPS-treated PMECs, the inhibitor of JNK phosphorylation did not affect caspase-3 activity. However, JNK phosphorylation may be involved in other processes in the response to LPS stimulation, and further investigation is required. Phosphorylated p38 MAPK not only downregulates antiapoptotic proteins such as Bcl-xl but also upregulates apoptotic proteins, including CHOP, p53 and cytochrome C^28,29^. In TEK/Capn4^−/−^ mice, p38 phosphorylation was significantly suppressed, indicating that the protective effect of Capn4 deficiency was related to decreased p38 phosphorylation. However, the precise crosstalk between p38 and calpain is currently unknown, and additional research is required to elucidate the potential mechanism.

Calpain activation-mediated endothelial cell apoptosis may impair vascular integrity, further increasing endothelial permeability, which directly contributes to organ failure. Increased vascular permeability in sepsis results in interstitial edema and fluid retention. Fluid overload and interstitial edema in renal microcirculation increase the diffusion distance of oxygen to target cells, leading to tubular cell injury^5^. In addition, activated endothelial cells upregulate expression of adhesion molecules and the release of additional proinflammatory mediators, which enhance leukocyte recruitment into the kidney during the pathological process of sepsis-induced AKI^30^. The recruited leukocytes, especially neutrophils, release proinflammatory mediators and molecules called damage-associated molecular patterns (DAMPs), which can directly damage tubular epithelial cells^31^. We found that endothelial-specific Capn4 knockout and calpastatin overexpression alleviated renal dysfunction in endotoxemic mice. However, the plasma BUN concentration did not significantly differ between wild-type and myeloid-specific Capn4 knockout mice. Together, these results indicate that calpain activation in the endothelial system plays a major role in LPS-induced renal dysfunction. Targeting endothelial calpain could thus be a potential therapeutic strategy for sepsis-induced AKI.

Moreover, we found that endotoxemic TEK/Capn4^−/−^ mice showed reduced production of NO and ROS, which contribute to endothelial dysfunction and microvascular injury in sepsis^32^. Previous studies found that activation of calpain-1 leads to IκB degradation, which is an essential step in the translocation and activation of nuclear factor-κB (NF-κB)^33^. Hence, a calpain inhibitor could prevent the expression of many NF-κB-dependent genes, including those encoding iNOS^34,35^. Since Capn4 encodes the small subunit of calpain-1 and calpain-2, our Capn4 knockout mice exhibited deficient activity of both calpain-1 and calpain-2. Decreased NF-κB activation might explain the reduced NO and ROS production in TEK/Capn4^−/−^ mice. Furthermore, we found that Capn4 knockout-induced iNOS downregulation might be related to inhibition of p38 phosphorylation, since phosphorylation of p38 also promotes the translocation and activation of NF-κB^36^. Notably, a deficiency in calpain activity decreased the expression of only iNOS and did not affect the protein expression of eNOS, although eNOS mRNA expression was decreased in TEK/Capn4^−/−^ mice. Under physiological conditions, NO synthesis is mediated by eNOS in the vasculature. eNOS activity is initiated in response to physical and chemical stimuli, functions as a homeostatic controller^37^ and is accepted to be protective against disease under physiological conditions. In contrast to eNOS, iNOS is not expressed in healthy states but is expressed under inflammatory conditions^38^. Moreover, the expression of iNOS has been proposed to be associated with disease states in the cardiovascular and renal systems^37,39^. Our results showed that targeting calpain could be a therapeutic approach for suppressing iNOS expression and further decreasing renal injury.

In summary, this study showed that knockout of endothelial calpain plays a protective role in LPS-induced AKI by inhibiting p38 phosphorylation to attenuate iNOS expression and further decrease endothelial apoptosis induced by NO/ROS overproduction.
